# Effect of Paroxetine Combined with Probiotics in Patients with Type 2 Diabetes Mellitus Complicated with Gastrointestinal Dysfunction and Liver Cancer

**DOI:** 10.1155/2021/4529915

**Published:** 2021-10-07

**Authors:** Yi Peng, Xingxia Yang, Yeju Wang

**Affiliations:** ^1^Department of Emergency, Wuhan Central Hospital Chinese Construction Third Engineering Bureau, Wuhan 430070, Hubei, China; ^2^Department of Infectious Disease, Shandong Binzhou Optimal Care Hospital, Binzhou 256606, Shandong, China; ^3^Department of Endocrine, Hanzhong Central Hospital, Hanzhong 723000, Shaanxi, China

## Abstract

**Background:**

To explore the effect of paroxetine combined with probiotics in patients with type 2 diabetes mellitus with gastrointestinal dysfunction and liver cancer and its effect on nutritional status.

**Materials and Methods:**

96 patients with type 2 diabetes mellitus combined with gastrointestinal dysfunction and liver cancer were selected as subjects from March 2018 to March 2021. They were randomly divided into control group and observation group, with 48 cases in each group. The control group was treated with probiotics, and the observation group was combined with paroxetine on the basis of the control group. After 4 weeks of treatment, the gastrointestinal mucosal function, nutritional status, Hamilton Depression Scale (HAMD) and Hamilton Anxiety Scale (HAMA) score, and the safety were compared between the two groups.

**Results:**

The levels of D-lactic acid, PCT, and endotoxin in the observation group were (1.75 ± 0.38), (4.39 ± 0.79), and (0.20 ± 0.06), respectively, which were significantly lower than those in the control group (2.69 ± 0.46), (7.84 ± 1.32), and (0.29 ± 0.08) (*P* < 0.05). Moreover, the nutritional status TP, ALB, Hb, PA, and TLC levels of the observation group were higher than those of the control group (*P* < 0.05). The HAMA and HAMD scores in the observation group were (5.76 ± 1.06) and (8.94 ± 1.26), respectively, which were significantly lower than those in the control group (10.69 ± 2.21) and (13.42 ± 2.34) (*P* < 0.05). However, there was no statistical significance in the incidence of nausea and vomiting, blurred vision, chest arthralgia, palpitation, anaesthesia, dizziness, and drowsiness between the two groups (*P* > 0.05).

**Conclusions:**

Paroxetine combined with probiotics could help to improve the gastrointestinal mucosal function of patients with type 2 diabetes mellitus complicated with gastrointestinal dysfunction and liver cancer, improve the nutritional status of patients, and reduce anxiety and depression, and the drug was safe and worthy of promotion and application.

## 1. Introduction

Liver cancer can be divided into primary liver cancer and secondary liver cancer. The former originates from the epithelial or mesenchymal tissue of the liver and the latter is caused by the metastasis of other tumors [1]. Epidemiological studies have confirmed that the incidence of primary liver cancer is increasing year by year, with an annual increase of over 700,000 cases, and primary liver cancer has now become the second leading cause of cancer-related deaths worldwide [2]. In China, about 383,000 people die of liver cancer every year, accounting for about 51% of the world's total. Liver cancer has become the fourth most common malignant tumor in China, with a poor prognosis and low survival rate [3, 4].

In recent years, with the change of people's lifestyles, diabetes has become one of the major chronic noncommunicable diseases affecting global health, and its prevalence has been increasing year by year [5]. Gastrointestinal dysfunction is one of the common chronic complications in patients with type 2 diabetes. It is more common in people with a long history of diabetes and older people and the clinical manifestations are heartburn, early satiety, postprandial discomfort, constipation or diarrhea, etc. [6]. Given the rapidly increasing incidence of liver cancer and type 2 diabetes, the number of people living with both diseases is also growing. Previous studies have shown that the mechanism of type 2 diabetes with gastrointestinal dysfunction and liver cancer has not yet been clarified, which might be related to autonomic nerve dysfunction, intestinal flora imbalance, smooth muscle damage, and gastrointestinal hormonal disorders, etc. [7]. Other studies have shown that dysregulation of the brain-gut axis can cause changes in intestinal movement and visceral perception, and patients are often accompanied by depression and psychological anxiety disorders, aggravating abnormalities and disorders of the digestive system [8, 9].

Probiotics are commonly used clinical gastrointestinal regulators, which can directly supplement the body's normal physiological bacteria and maintain the normal intestinal flora [10, 11]. Paroxetine is a commonly used clinical antidepressant, which can selectively inhibit 5-hydroxytryptamine (5-HT) transporter, block the reuptake of 5-HT by the presynaptic membrane, and exert an antidepressant effect [12]. This present study was aimed to explore the effect of paroxetine combined with probiotics in patients with type 2 diabetes mellitus complicated with gastrointestinal dysfunction and liver cancer. The report was as follows.

## 2. Materials and Methods

### 2.1. Clinical Data

Ninety-six patients with type 2 diabetes mellitus combined with gastrointestinal dysfunction and liver cancer were selected from March 2018 to March 2021 at Wuhan Central Hospital Chinese Construction Third Engineering Bureau, Wuhan, Hubei, China, and they were randomly divided into observation group and control group, with 48 cases in each group. Control group: 31 males and 17 females, age (41–73) years old, average (60.39 ± 5.61) years old, body mass index (BMI) (18–29) kg/m^2^, average (23.51 ± 3.49) kg/m^2^, duration of type 2 diabetes (1–15) years, average (9.15 ± 0.95) years, duration of gastrointestinal disorders (1–6) years, average (3.23 ± 0.51) years, and complications: 3 cases of hypertension, high 6 cases of lipemia. Observation group: 29 males and 19 females, aged (42–74) years old, average (60.46 ± 5.68) years old, BMI (17–30) kg/m^2^, average (23.68 ± 3.54) kg/m^2^, type 2 diabetes course (1–16) years, average (9.21 ± 0.99) years, gastrointestinal dysfunction course (1–7) years, average (3.32 ± 0.58) years, and complications: 4 cases of hypertension, 5 cases of hyperlipidemia. There was no statistically significant difference in general information between the two groups of patients (*P* > 0.05), and they were comparable. The study was approved by the ethics committee of Wuhan Central Hospital Chinese Construction Third Engineering Bureau, Wuhan, Hubei, China, and informed consent was obtained from the patients.

### 2.2. Inclusion and Exclusion Criteria

Inclusion criteria: (1) meeting the diagnostic criteria for type 2 diabetes, with different degrees of gastrointestinal dysfunction. (2) Meeting the diagnostic criteria for liver cancer, and diagnosed by pathological examination. (3) Meeting paroxetine, probiotics drug therapy indications; no history of drug allergy. (4) Completing baseline and follow-up data.

Exclusion criteria: (1) severe liver and kidney dysfunction or taking glucocorticoids or immune enhancers in the past 3 months. (2) Mental disorders, organic diseases, or blood system diseases. (3) Cognitive dysfunction; abnormal blood coagulation function.

### 2.3. Method

After admission, both groups were given symptomatic and supportive treatment intervention. Individuals with type 2 diabetes should eat low-salt and low-fat foods and fresh vegetables and fruits and strengthen the rehabilitation exercise according to the patients' recovery. Control groups were treated with probiotics. Patients took 2 capsules of probiotics (*Bifidobacterium*, *Enterococcus,* and *Lactobacillus acidophilus*) capsules (Shandong Hengjia Biotechnology Co., Ltd., sc11337082906741) each time, orally, 3 times a day. According to the patient's tolerance and response, the drug dose was increased appropriately according to the drug instructions. Observation groups were combined with paroxetine treatment on the basis of the control group. The initial dose of paroxetine (Zhejiang Huahai Pharmaceutical Co., Ltd., National Medicine Standard H20031106, specification: 20 mg) was 5 mg each time, orally, and the drug dose was increased every 5 days (the drug dose is 20 mg), and other psychotropic drugs were avoided during treatment. Effects for each patient were evaluated after 4 weeks of treatment.

### 2.4. Observation Indicators

(1) Gastrointestinal mucosal function: the modified enzymatic spectrophotometry was used to determine the D-lactic acid level before treatment and 4 weeks after treatment. A quantitative immunoluminescence method was used to determine the patient's procalcitonin (PCT) level [13]. (2) Nutritional status: an automatic biochemical analyzer was used to determine the total protein (TP), hemoglobin (Hb), prealbumin (PA), total lymphocyte count of the patients (TLC), and serum albumin (ALB) levels [14]. (3) Psychological fluctuations: the Hamilton Depression Scale (HAMD) and Hamilton Anxiety Scale (HAMA) were used to evaluate the psychological fluctuations of the patients before and 4 weeks after treatment. The lower the score, the better the effect [15, 16]. (4) Security: the incidence of nausea and vomiting, blurred vision, chest palpitations, palpitation, akathisia, dizziness, and drowsiness during the two groups was recorded.

### 2.5. Statistical Analysis

SPSS24.0 software was used to measure the statistical data. The count data were analyzed by *χ*2 test, expressed by *n* (%). D-Lactic acid, PCT, endotoxin, and other measurement data were in accordance with the normal distribution and analyzed by *t* test, expressed by ($\bar{x}\pm s$). *P* < 0.05 was considered as statistically significant.

## 3. Results

### 3.1. Comparison of Gastrointestinal Mucosal Function between the Two Groups

The function of gastrointestinal mucosa before treatment in the two groups was not statistically significant (*P* > 0.05). The gastrointestinal mucosal function of the two groups was improved 4 weeks after treatment. The levels of D-lactic acid, PCT, and endotoxin in the observation group were lower than those in the control group (*P* < 0.05), as shown in Table 1.

### 3.2. Comparison of Nutritional Status between the Two Groups

The nutritional status of the two groups before treatment was not statistically significant (*P* > 0.05). After 4 weeks of treatment, the nutritional status of the two groups was significantly improved, and the nutritional status of the observation group was higher than that of the control group (*P* < 0.05), as shown in Table 2.

### 3.3. Comparison of Psychological Fluctuations between the Two Groups

Psychological fluctuations before treatment in the two groups were not statistically significant (*P* > 0.05). Four weeks after treatment, the psychological symptoms of the two groups were significantly improved (*P* < 0.05); the HAMA and HAMD scores of the observation group were lower than those of the control group (*P* < 0.05), as shown in Table 3.

### 3.4. Comparison of the Safety of the Two Groups

The incidence of nausea and vomiting, blurred vision, chest palpitations, akathisia, and dizziness and drowsiness during the treatment of the two groups was not statistically significant (*P* > 0.05), as shown in Table 4.

## 4. Discussion

With the change of lifestyle, diabetes has become the third most harmful disease to human health after malignant tumors and cardiovascular and cerebrovascular diseases, and its morbidity and mortality have been on the rise year by year. In 2013, 3.82 billion people were diagnosed with diabetes, which is expected to increase to 5.92 billion worldwide [17]. Yancik et al. confirmed that the 30-month mortality of breast cancer patients with diabetes was 76% higher than that of patients without diabetes after adjusting for age and staging [18]. At the same time, another clinical study showed that after adjusting for age, sex, and stage, the mortality rate of colon cancer patients with diabetes increased by 37% [19]. A randomized adjuvant chemotherapy study conducted by Pechlivanis et al. also confirmed that 287 colon cancer patients with diabetes mellitus had a 42% increased risk of death and a 21% increased rate of tumor recurrence [20]. Previous studies have shown that the intestine is the “second brain” of humans. The continuous stress response will cause mental and psychological abnormalities, which will cause hyperesthesia in the intestines and internal organs in patients with type 2 diabetes and gastrointestinal dysfunction. Meanwhile, the increased sensitivity threshold of the intestine will cause intestinal spasms and abnormal motility [21, 22]. Therefore, the search for specific drugs for early prediction of liver cancer with diabetes has important clinical significance.

In recent years, paroxetine combined with probiotics has been used in patients with type 2 diabetes with gastrointestinal dysfunction and liver cancer, and the effect is ideal [23]. In this study, the levels of D-lactic acid, PCT, and endotoxin in the observation group were lower than those in the control group (*P* < 0.05) 4 weeks after treatment, indicating that paroxetine combined with probiotics can improve gastrointestinal dysfunction in patients with type 2 diabetes and liver cancer, which is beneficial to the recovery of patients. Probiotics are commonly used clinical gastrointestinal regulators, which can directly supplement the normal physiological bacteria of the body and maintain the normal intestinal flora [24]. Moreover, probiotics can inhibit and remove potentially harmful bacteria in the intestines and can regulate the disorder of the body's microecological balance [10]. Previous studies have shown that probiotics can help patients rebuild and protect the gastrointestinal flora barrier, thereby organizing the invasion of foreign pathogenic bacteria, inhibiting endotoxins produced by harmful bacteria, reducing the translocation of bacteria and endotoxins in the intestines, maintaining the structural integrity of the gastrointestinal tract and enhancing the local defense ability of the gastrointestinal mucosa [25].

In this study, the nutritional status TP, ALB, Hb, PA, and TLC levels of the observation group were higher than those of the control group 4 weeks after treatment (*P* < 0.05). The HAMA and HAMD scores of the observation group were lower than those of the control group 4 weeks after treatment (*P* < 0.05), indicating that paroxetine can improve the psychological fluctuations of patients with type 2 diabetes and gastrointestinal dysfunction and improve the nutritional status of patients. Clinically, paroxetine combined with probiotics can be used in patients with type 2 diabetes with gastrointestinal dysfunction and liver cancer, which can give play to the advantages of different drugs. Moreover, the combined use of the drugs is safer, which helps to improve patient tolerance and compliance. In addition, the incidence of nausea and vomiting, blurred vision, chest palpitations, akathisia, and dizziness and drowsiness during the treatment of the two groups was not statistically significant (*P* > 0.05), indicating that paroxetine combined with probiotics is safer in the treatment of type 2 diabetes with gastrointestinal dysfunction and liver cancer.

## 5. Conclusion

Paroxetine combined with probiotics can help improve gastrointestinal mucosal function in patients with type 2 diabetes complicated with gastrointestinal dysfunction and liver cancer, help improve the nutritional status of patients, and reduce anxiety and depression. The drug is safe and worthy of promotion and application.

## Figures and Tables

**Table 1:** Comparison of gastrointestinal mucosal function between the two groups ($\bar{x}\pm s$).

Group		D-Lactic acid (mmol/L)	PCT (ug/L)	Endotoxin (EU/mL)
Observation group (*n* = 48)	Before treatment	3.10 ± 0.53	10.43 ± 2.14	0.33 ± 0.08
	Four weeks after treatment	1.75 ± 0.38^#^^*∗*^	4.39 ± 0.79^#^^*∗*^	0.20 ± 0.06^#^^*∗*^

Control group (*n* = 48)	Before treatment	3.12 ± 0.55	10.45 ± 2.16	0.35 ± 0.10
	Four weeks after treatment	2.69 ± 0.46^*∗*^	7.84 ± 1.32^*∗*^	0.29 ± 0.08^*∗*^

^#^ *P* < 0.05 vs control group; ^*∗*^*P* < 0.05 vs before treatment.

**Table 2:** Comparison of nutritional status between the two groups ($\bar{x}\pm s$).

Group		TP (g/L)	ALB (g/L)	Hb (g/L)	PA (mg/L)	TLC (×10^9^/L)
Observation group (*n* = 48)	Before treatment	50.39 ± 2.63	28.51 ± 4.69	97.35 ± 6.73	144.39 ± 12.59	1.16 ± 0.21
	Four weeks after treatment	64.53 ± 4.59^#^^*∗*^	35.63 ± 5.71^#^^*∗*^	119.69 ± 10.69^#^^*∗*^	191.56 ± 14.66^#^^*∗*^	1.82 ± 0.53^#^^*∗*^

Control group (*n* = 48)	Before treatment	50.41 ± 2.64	28.52 ± 4.70	97.37 ± 6.75	145.41 ± 12.62	1.17 ± 0.23
	Four weeks after treatment	58.59 ± 4.31^*∗*^	30.59 ± 5.36^*∗*^	105.63 ± 8.52^*∗*^	158.96 ± 13.48^*∗*^	1.35 ± 0.51^*∗*^

^#^ *P* < 0.05 vs control group; ^*∗*^*P* < 0.05 vs before treatment.

**Table 3:** Comparison of psychological fluctuations between the two groups (points $\bar{x}\pm s$).

Group	Case	HAMA		HAMD	
		Before treatment	Four weeks after treatment	Before treatment	Four weeks after treatment
Observation group	48	24.69 ± 3.57	5.76 ± 1.06^#^	28.63 ± 4.35	8.94 ± 1.26^#^
Control group	48	24.72 ± 3.59	10.69 ± 2.21^#^	28.66 ± 4.38	13.42 ± 2.34^#^
*T*	—	0.583	12.593	1.448	10.336
*P* value	—	0.409	<0.001	0.925	<0.001

^#^ *P* < 0.05 vs before treatment.

**Table 4:** Comparison of the safety of the two groups (*n* (%)).

Group	Case	Sick and vomiting	Blurred vision	Chest palpitations	Cannot sit still	Dizziness and drowsiness	Incidence
Observation group	48	0 (0.00)	1 (2.08)	0 (0.00)	1 (2.08)	1 (2.08)	3 (6.25)
Control group	48	1 (2.08)	1 (2.08)	1 (2.08)	0 (0.00)	1 (2.08)	4 (8.3)
*x* ^2^	—						0.154
*P* value	—						0.695

## Data Availability

The datasets used and/or analyzed during the present study are available from the corresponding author on reasonable request.
